# Neanderthal and Denisovan Glutamate Dehydrogenase 2 Evolution and Clinical Significance

**DOI:** 10.3390/ijms26094322

**Published:** 2025-05-01

**Authors:** Yulia A. Aleshina, Lev G. Zavileyskiy, Vasily A. Aleshin

**Affiliations:** 1 Martsinovsky Institute of Medical Parasitology, Tropical and Vector Borne Diseases, Sechenov First Moscow State Medical University, 119435 Moscow, Russia; vjulia94@gmail.com; 2 Faculty of Bioengineering and Bioinformatics, Lomonosov Moscow State University, 119234 Moscow, Russia; 3 Belozersky Institute of Physicochemical Biology, Lomonosov Moscow State University, 119234 Moscow, Russia; 4 Department of Biochemistry, Sechenov First Moscow State Medical University, 119048 Moscow, Russia

**Keywords:** glutamate dehydrogenase, *GLUD2*, human evolution, GLUD1P3, miR-27, Parkinson’s disease, Denisovan, Neanderthal

## Abstract

Mammalian glutamate dehydrogenase (GDH) is an indispensable metabolic enzyme. GDH duplication has led to the presence of two paralogs, GDH1 and GDH2, in apes. Multiple GDH pseudogenes are also present in the human genome. The novel GDH2, supposed to be a target of positive selection, differs from GDH1 in regulation and is believed to be tightly linked to brain development. Although the differences of modern human GDH2 from GDH2 of other apes have been studied, the evolution of ancient human GDH2 remains a blank space. The goal of this work was to elucidate GDH2 evolution in the genus *Homo* using the accumulated data on the ancient genomes with high coverage—three Neanderthal and one Denisovan genome. Such analysis clarifies the difference between GDH2 of the last common ancestor of humans and chimpanzees and all *Homo* to be in M468L substitution, localized in the regulatory “antenna” region of the protein. A few novel missense mutations have been found in Denisovan and Altai Neanderthal GDH2, namely R76H, present in both genomes, and Denisovan-specific T154P, I358L, and S498A substitutions. Another mutation, R352K, has likely occurred independently in modern humans and later Neanderthals. The potential impact of these mutations was estimated using GDH2 structural data and evidence from contemporary medical data. All substitutions are supposed to be benign, with only the S498A GDH2 substitution connected to Parkinson’s disease with late onset. Additionally, the ancient genomes were revealed to have all GDH pseudogenes present in modern humans, including the RNA-coding ones. The *GLUD1P3* RNA expression was found to correlate negatively with GDH1 in human tissues. A possible regulatory role has been proposed, and the *GLUD1P3* RNA sequence identity in all the studied human genomes suggests its conservation in the genus *Homo*.

## 1. Introduction

The human genome encodes two functional copies of glutamate dehydrogenase (GDH), namely *GLUD1* and *GLUD2*, as a result of relatively recent gene duplication [1]. The *GLUD2* gene is found in apes/hominoids, while other mammals have only the *GLUD1* gene. *GLUD2* appearance and further evolution approximately coincide with the period of increase in size and complexity of the primate brain [2]. Thus, the study of the specific function of glutamate dehydrogenase 2 (GDH2) encoded by *GLUD2* is highly important for understanding human evolution.

The *GLUD2* gene emerged due to a retrotransposition to the X chromosome less than 23 million years ago [2]. A large number of copies of various genes were generated at that time in primates due to a burst of retrotranspositions, which began ∼40–50 million years ago [3]. At least five more GDH pseudogenes are present in the human genome, indicating multiple episodes of *GLUD1* retrotranspositions [4]. Of all these copies, only *GLUD2* remains a protein-coding gene. GDH2 retained its enzymatic function, indicating an evolutionary benefit to *GLUD2* carriers.

Found in all living organisms [5], GDH is an indispensable enzyme linking metabolism of carbon and nitrogen. Mammalian GDH, including GDH2, catalyzes a reversible oxidation of L-glutamate. The latter is the main excitatory neurotransmitter in the brain, and the function of GDH2 is believed to be tightly linked to brain development in apes [4,6,7]. In addition to enzymatic function, GDH may form filamentous and lamellar supramolecular structures [8,9], which may be relevant for specific functions of GDH, such as *Drosophila melanogaster* testicular *Bb8* GDH [10]. A comparison of the GDH2 and *Bb8* GDH, both expressed the most in testes, suggests a potential evolutionary benefit of GDH2 due to its testes-specific function, which is studied rather poorly. To date, the main GDH2 difference from GDH1 is considered to be in its more efficient allosteric regulation and thus more efficient response to metabolic alterations of ADP/ATP(GTP) levels, which are among the main regulators of GDH2 [2,11,12,13].

Despite GDH2 allosteric regulation being studied rather well, there are substantial blank spaces in understanding the GDH2 role and evolution, as has been shown in our recent review [4]. One such blank space is the evolution of GDH2 in the human lineage. Its understanding may elucidate more details on the function of this enzyme because of its link to the evolution of the brain in apes. Of note, other functions of GDH2 may be of no less importance [4,13,14]. Particularly, the importance of GDH2 in testes is supported by data from a patient with a *GLUD2* nonsense mutation causing oligospermia [15]. Thus, here we aimed at analyzing accumulated data on the genomes of ancient humans, revealing their GDH2 sequences, and putting them into the context of GDH2 evolution in apes. Such analysis added a substantial piece of knowledge to the overall analysis of GDH2 evolution recently prepared by us [4]. The presence of Denisovan- and Neanderthal-like variants in the human population and their clinical significance were further evaluated. We also checked the ancient genomes for the presence of GDH pseudogenes, some of which are known to be RNA-coding [16]. Special attention was paid to *GLUD1P3* due to an established correlation of its RNA expression with GDH1 mRNA in modern humans.

## 2. Results

### 2.1. Phylogenetic Analysis of Neanderthal and Denisovan GLUD2 Sequence

**Figure 1:**
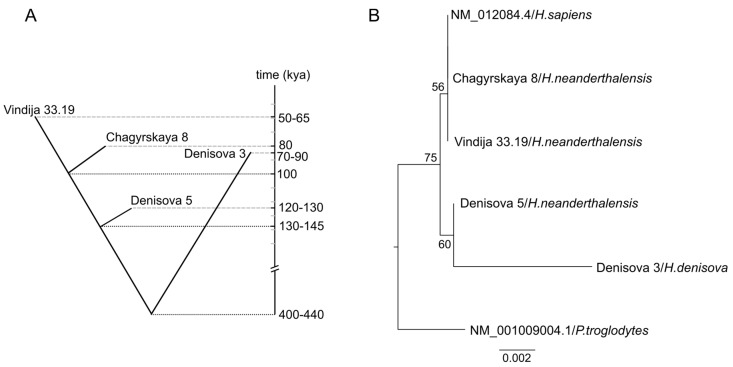
Evolutionary relationships of ancient humans and their GLUD2 gene. (**A**)—The evolutionary relationships of high-coverage genomes (>27-fold) of ancient humans. The estimates of genome ages and population split time estimates are indicated by dashed and dotted lines, respectively, according to [19,20]. (**B**)—Maximum likelihood phylogenetic tree of *GLUD2* mRNA in ancient humans with high-coverage genomes. The tree was rooted by an outgroup (chimpanzee, *Pan troglodytes*). The tree bar indicates the number of substitutions per site. The ultrafast bootstrap supports are indicated at the tree nodes.

In order to infer the evolutionary history of *GLUD2* with accumulated data on archaic humans, we considered the genomes with high quality (coverage over 27-fold) (Table S1). To date, four genomes of ancient humans have been sequenced with high coverage: one genome of Denisovan found in Denisova cave in the Altai Mountains in southern Siberia (*Denisova 3*) [17], and three Neanderthal genomes—*Denisova 5* (or Altai Neanderthal) [18], *Chagyrskaya 8* [19], and *Vindija 33.19* [20]. According to molecular dating estimates, the ancestors of modern humans split from the common ancestor of Neanderthals and Denisovans between 553 and 589 kya (1000 years ago) [18]. The most common recent ancestor of *Denisova 3* and Neanderthals lived 400–440 kya (Figure 1A) [20]. *Denisova 5* split from populations ancestral to other Neanderthals 130–145 kya [20]. *Chagyrskaya 8*, found in Chagyrskaya cave in the northwest Altai Mountains, is more closely related to west European Neanderthal *Vindija 33.19* than to *Denisova 5* (Altai Neanderthal). The population split of *Chagyrskaya 8* and *Vindija 33.19* occurred ~80–100 kya [19]. The Neanderthals represented by these two genomes will be further referred to as younger Neanderthals, similarly to [21]. The phylogeny of *GLUD2* mRNA sequences from modern and archaic humans did not coincide with the species tree, although the bootstrap supports of the tree branches were relatively low and did not exceed 75% (Figure 1B). *GLUD2* sequences of modern human, the *Vindija 33.19,* and *Chagyrskaya 8* Neanderthal individuals were identical to each other. The *GLUD2* sequence of *Denisova 5* (Altai Neanderthal) was more similar to *GLUD2* of Denisovan *Denisova 3* than to other Neanderthal sequences. Noteworthy, the length of the Denisovan branch was much longer compared to other human sequences, indicating more substitutions in this sequence (Figure 1B).

Analyzing the length of the Denisovan branch and the potential effect of the accumulation of substitutions upon evolution, one should also take into account the approximate age of the samples. On one hand, more recent samples had less time for the accumulation of substitutions. On the other hand, older samples are more prone to DNA damage. The Denisovan (*Denisova 3*) lived approximately 80 kya, which is similar to the “*Chagyrskaya 8*” Neanderthal, ~30 ky (1000 years) after the Altai Neanderthal (*Denisova 5*) and ~30 ky before *Vindija 33.19* [19]. Thus, *Denisova 3* is not the youngest and not the oldest one among available samples, and none of these criteria can explain that many substitutions.

### 2.2. Substitutions in Denisovan GLUD1 Illustrate the Level of DNA Degradation

In order to check the hypothesis of enhanced GLUD2 evolution in Denisovans, we analyzed the *GLUD1* sequences. Unlike *GLUD2*, *GLUD1* is considered to be a target for purifying selection [2]. Thus, *GLUD1* sequences are expected to be identical or at least very similar among all human species. However, multiple sequence alignment (Supplementary Figure S1) revealed ten substitutions in Denisovan *GLUD1*. Since these substitutions are most likely due to the ancient DNA degradation, we have divided them into subgroups according to their potential to be caused by deamination of adenine to hypoxanthine (transition type 1 or TS1) and the deamination of cytosine or 5-methyl cytosine to uracil or thymine (TS2), respectively [22]. Only one substitution could not be explained by DNA degradation (Table 1). However, this c.909A>C mutation is synonymous, causing no amino acid (Thr303) substitution in GDH1. Thus, these data are in good accordance with the known high conservation of the *GLUD1* gene.

As a result, analysis of the *GLUD1* sequence, which is identical in terms of its length to *GLUD2*, indicates that approximately nine substitutions of Denisovan *GLUD2* could be due to the spontaneous DNA degradation. As for other genomes, their substitutions are more likely to be caused by mutations rather than DNA degradation.

### 2.3. Exclusion of GLUD2 Substitutions Caused by DNA Degradation

Due to the presence of substitutions in Denisovan *GLUD1*, which most likely were caused by DNA degradation, a detailed examination of substitutions in *GLUD2* was needed. From the equal length of *GLUD1* and *GLUD2* coding sequences, one could expect approximately nine substitutions in Denisovan *GLUD2* also caused by DNA degradation, and almost none such substitutions in the Neanderthal sequences—similarly to the results of *GLUD1* analysis (Table 1, Figure S1).

According to the multiple sequence alignment of *GLUD2*, there are 13 substitutions in the Denisovan gene, which do not correspond to the ancestral genome (Figure S2). These substitutions are summarized in Table 2.

Thus, the expectations of nine substitutions in the *GLUD2* of the Denisovan genome agree well with the eight substitutions belonging to either TS1 or TS2 and absent in other genomes (Table 2, Figure S2). Still, there are four substitutions likely caused by mutations, of which two are synonymous (c.94C>A and c.123G>T encode Arg32 and Ser41, respectively). The two remaining mutations are c.460A>C and c.1492T>G corresponding to amino acid substitutions T154P and S498A, respectively. In addition, the Denisovan *GLUD2* sequence has c.1072A>C substitution causing the I358L mutation, which is also found in chimpanzee branch, while all other Hominoidea have Ile in the corresponding GDH2 position [4]. The ancestral state reconstruction analysis suggested that c.1072A>C mutation independently occurred in Denisovan and Chimpanzee GDH2.

### 2.4. Genetic Insights into GDH2 Evolution from Common Mutations of Altai Neanderthal and Denisovan

**Figure 2:**
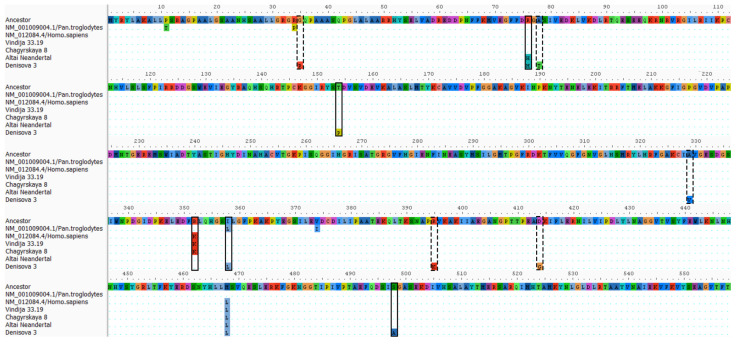
Multiple sequence alignment for GDH2 sequences from chimpanzees, modern and archaic humans. The ancestral sequence was inferred using the maximum likelihood method [23] under the Kimura 2-parameter model [24] implemented in MEGA11 [25]. Dots indicate amino acids that are identical to the ancestral sequence. The alignment was visualized in AliView v1.28 software [26]. Columns with substitutions in *Denisova 3* and/or Altai Neanderthal, which likely occurred due to mutations, are marked with black rectangles. The substitutions, which are likely the result of DNA degradation, are marked with dashed rectangles.

The new data on Denisovan and Neanderthal GDH2 sequences revealed that all GDH sequences belonging to the human clade possess c.1402A>C mutation, resulting in M468L, which differentiates humans from all other Hominoidea. Thus, of the two mutations that differentiate modern humans from their common ancestor with chimpanzees, namely M468L and R352K, the first one is also found in Neanderthals and Denisovans (Figure 2). As for R352K, this substitution caused by c.1055G>A is specific to modern humans and younger Neanderthals, while Denisovans and the most ancient Altai Neanderthals possess Arg352, which is common for most other apes. Thus, ancient genomic data reveals that of the two *H.sapiens*-specific amino acid substitutions, M468L was the first one, differentiating the genus *Homo* from other apes, while the R352K substitution was the last mutation of GDH2 in modern humans. The latter is also present in younger Neanderthals. Of note, R352K is the reversal mutation, and GDH2 of gibbons also has Lys residue in the corresponding position, while most Hominidae have Arg352 [4].

Additionally, Altai Neanderthal and Denisovan have a c.227G>A missense mutation causing R76H substitution. The latter mutation has not been observed before in other apes.

### 2.5. Structural Analysis of the Novel GDH2 Mutations Found in Altai Neanderthal and Denisovan Genomes

**Figure 3:**
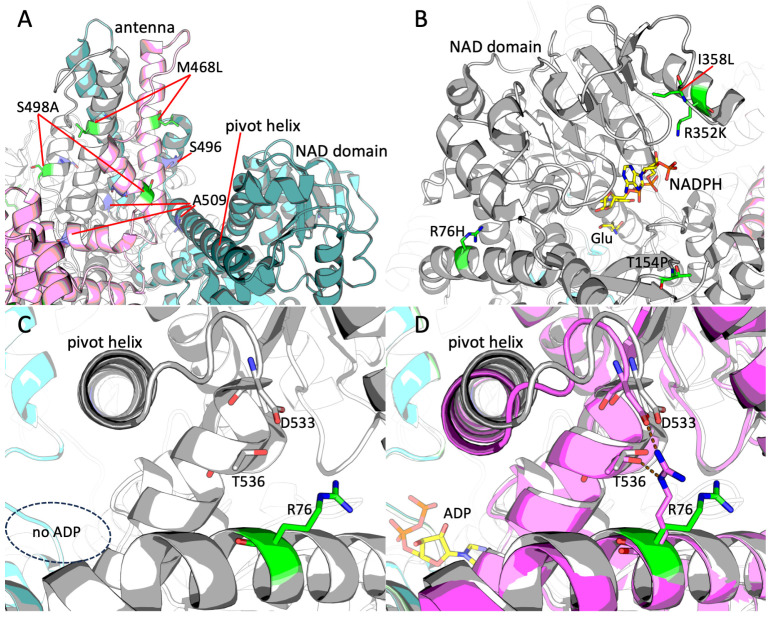
Localization of the novel substitutions in the structure of human GDH2. (**A**)—Structural localization of the mutated residues S498A and M468L in the antenna region of GDH2 (PDB ID: 6G2U, [27]). The S498A and M468L residues are colored in green. The Ser496 and Ala509 residues important for the evolutionary development and function of GDH2 are colored in blue. Three different subunits are colored in pink, light grey, and teal. (**B**)—Structural localization of the mutated residues I358L, R352K, T154P, and R76H in the NAD domain of GDH2. The mutated residues are colored green. Active site is indicated with the help of an abortive complex of substrates glutamate and NADPH colored in yellow. (**C**)—A closer look at the localization of Arg76 residue mutated in Altai Neanderthal and Denisovan. Thr536 and Asp533 residues are revealed. Position of the ADP site close to the pivot helix is shown with a dashed oval. (**D**)—A merge of the GDH structures with ADP (GDH shown in magenta, PDB ID: 8AR7, [28]) and without ADP (GDH shown in grey as in (**C**), PDB ID: 6G2U). The difference in positions of the pivot helix and the Arg76 residue is shown. If applicable, the interaction of Arg536 with Thr536 and Asp533 residues is shown with dashed lines. ADP is shown in yellow.

As discussed above, the Leu468 residue is specific for the genus *Homo*, while all other apes have Met residue in this position. Structural localization of this mutation reveals it in the antenna region, particularly in its long helix (Figure 3A). Another mutation localized in this region is S498A, found in the Denisovan genome. This residue takes place in the small helix of the antenna (Figure 3A). Although the effects of these particular mutations are not clear, the region is important for allosteric regulation of GDH. Particularly, the two residues whose emergence in the evolution of GDH2 is considered the most important, Ser496 and Ala509, are located in this region (Figure 3A). Mutations of these residues are responsible for GDH2’s lower inhibition by GTP (Ser496) and protein stability (Ala509). Position of Leu468 may suggest its influence on the interaction between the two helices of the antenna (Figure 3A). As for the Denisovan-specific S498A substitution, its localization not far from the Ser496 residue in the same small helix of the antenna indicates a potential effect on allosteric regulation of the enzyme.

The other mutations found in the genus *Homo*, namely I358L, R352K, T154P, and R76H, take place not far from the NAD domain of GDH2 (Figure 3B). Of these mutations, R352K independently occurred in ancestors of modern humans and younger Neanderthals. The residue is involved in the binding of substrate NADP(H) (Figure 3B). Next to it, there is a Denisovan-specific mutation, I358L (Figure 3B); however, it does not participate directly in the substrate binding but only influences the hydrophobic core of this part of the NAD domain. Similarity of Ile and Leu residues suggests only a moderate potential effect on the enzyme properties. Another T154P mutation is also specific for Denisovans. The residue is in proximity to the NAD(P)(H) site and is localized on the protein surface (Figure 3B). It belongs to a loop; thus, the Pro residue cannot disrupt the secondary structure of the protein. Still, the mutation can influence the flexibility of the loop and potential interactions of the residue.

Last but not least, the novel mutation R76H found simultaneously in Altai Neanderthal and Denisovan GDH2 is localized in the first α-helix of “mature” protein (i.e., the protein without N-terminal peptide possessing mitochondrial targeting signal) (Figure 3B–D). Arg76 takes its place on the protein surface close to the last C-terminal helix of GDH, particularly its residue Thr536 (Figure 3C,D). Some parts of GDH, such as the pivot helix, are especially flexible upon allosteric regulation of the enzyme. The pivot helix precedes the C-terminal helix, and the occupancy of the ADP site influences its position (Figure 3C,D). Importantly, the loop following the pivot helix contains Asp533 residue, which is also located close to Arg76 (Figure 3C,D). Comparison of the GDH structures without and with ADP bound elucidates the difference in conformation of Arg76. In the presence of ADP, this residue forms two bonds with Asp533 and Thr536 residues (Figure 3D). The substitution of Arg76 a shorter His residue observed in Altai Neanderthal and Denisovan should prevent the formation of an ionic bond with Asp533, while maintaining the possibility of forming a hydrogen bond with Thr536.

### 2.6. Potential Clinical Singificance of Neanderthal and Denisovan GDH2 Variants

Novel variants of GDH2 possessing mutations specific to Neanderthal and Denisovan humans might also be found in modern humans. Such variants could occur either by mutations de novo or as a part of Neanderthal or Denisovan DNA in our genome. The analysis of clinical relevance of the mutations can provide a better understanding of the evolution of ancient humans and the role of GDH2 in it. The data on such found mutations is summarized in Table 3.

There are five variants, all of which occur as single-point mutations. The variant c.227G>A, causing R76H substitution both in Altai Neanderthal and Denisovan humans, is considered to be a somatic mutation found in colon adenocarcinoma. The residue is localized in the N-terminal helix and is likely involved in allosteric regulation of GDH2 (Figure 3C,D). The variant is proposed to be moderately pathogenic. No data on its allele frequency and occurrence in the population is available except for somatic mutations (Table 3).

The c.460A>C variant causing T154P substitution in Denisovan is rather frequent in modern humans, with an approximate frequency of 0.02%. The substitution occurs not far from the active site (Figure 3B). However, in accordance with its frequency, the variant is likely benign and no data on its clinical significance is available.

The mutation c.1072A>C found in Denisovan and causing I358L substitution in the NAD domain of GDH2 has a frequency of approximately 0.00008% (Table 3). Despite such a rare occurrence in the population, this synonymous mutation is considered benign, and no data on its clinical significance can be found.

Another reverse mutation c.1402C>A, causing L468M substitution, was found (Table 3). The Leu468 residue is localized in the antenna (Figure 3A) and is specific to all humans, however Met468 variant is present in most Hominoidea. No data on its frequency or clinical significance is available.

Finally, the c.1492T>G mutation found in Denisovan GDH2 and causing S498A substitution in the antenna region (Figure 3A) has a frequency of approximately 3% in the modern human population (Table 3). This variant is responsible for the predisposition to Parkinson’s disease with late onset [29]. Thus, despite its pathogenicity, the mutation is not only among the most frequent GDH2 variants in the modern human population, but is also found in the Denisovan genome.

Distribution analysis of allele frequencies among modern populations was enabled for the three substitutions found in the Denisovan genome (T154P, I358L, and S498A). Such analysis indicates that T154P and S498A variants are the most frequent in the African/African American genetic ancestry group and absent or are the least frequent in the East Asian group (Table S2). Vice versa, I358L variant is found only in the East Asian group. However, there is limited availability of public data from the areas of Denisovan inhabitance which may affect such analysis. Still, from the allele frequencies (Table S2) it can be proposed [30,31], that only the I358L variant may result from an admixture of Denisovan DNA. Noteworthy, local populations may possess higher allele frequencies. For example, to date, the highest frequency of the S498A variant in the world is found in the Daghestan region (Table S2) [32].

**Table 3:** Availability of the missense GDH2 variants similar to Neanderthal or Denisovan GDH2 in modern humans and their potential medical significance. Data from the ClinVar [33], COSMIC [34], NCBI dbSNP [35], Ensembl [36], gnomAD [37], and Uniprot [38] databases are used for the evaluation of potential pathogenicity. Allele frequency is indicated as total allele frequency from gnomAD. The medical condition is written as submitted to a database, if available. The structural position of the mutations is provided as assessed in this work.

Variant	Allele Frequency	Submitted Condition	Structural Data	Database
R76H (c.227G>A)	–	Somatic, Colon Adenocarcinoma	N-terminal helix	COSMIC
T154P (c.460A>C)	0.0002231	–	Entrance to the active site	gnomAD, dbSNP
I358L (c.1072A>C)	8.268 × 10^−7^	–	NAD domain	gnomAD
L468M (c.1402C>A)	–	–	Antenna, large helix	Ensembl, Uniprot
S498A (c.1492T>G)	0.02618	Parkinson’s disease, late-onset	Antenna, small helix	gnomAD, ClinVar

### 2.7. Availability of GDH Pseudogenes in Neanderthal and Denisovan Genomes and Their Plausible Role

**Figure 4:**
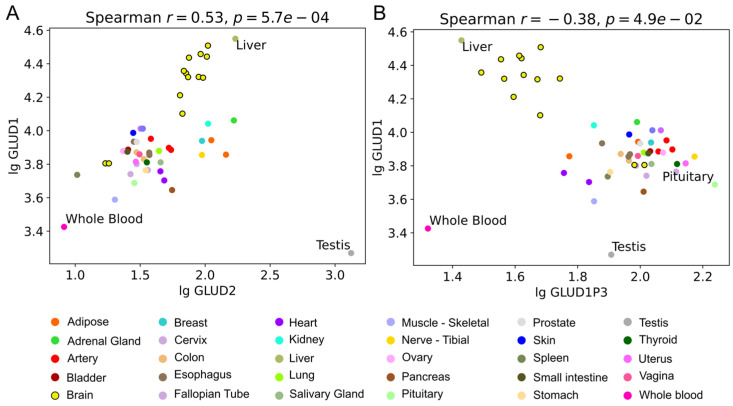
Correlations between expression of GDH genes and pseudogenes. The expression of *GLUD1*, *GLUD2,* and GDH pseudogenes in various human tissues was analyzed with Spearman correlations using GTEx bulk RNA sequencing data. The data corresponding to cell cultures were excluded from the analysis. Two significant (*p* < 0.05) correlations were found, namely (**A**) correlation between *GLUD1* and *GLUD2* and (**B**) correlation between *GLUD1* and *GLUD1P3* pseudogene. Samples corresponding to the brain areas are encircled with black lines, other tissues are colored as indicated in the legend. Tissues with minimum and maximum values are revealed using text labels.

There are many GDH pseudogenes in the human genome. Although *GLUD1* pseudogenes are not protein-coding, RNA can be transcribed from some of them. Sequence similarity of these RNA to GDH1 and GDH2 implies their potential involvement in GDH regulation. Using Genotype-Tissue Expression (GTEx) database, we have identified a positive correlation between expression of GDH1 (*GLUD1*) and GDH2 (*GLUD2*) (r = 0.53, *p* < 0.001) and a negative correlation between expression of *GLUD1* and *GLUD1P3* (r = −0.38, *p* = 0.049) (Figure 4B). No other significant correlations have been found, while a trend (0.05 < *p* < 0.1) for correlation between *GLUD2* and *GLUD1P3* was observed (r = −0.35, *p* = 0.078).

Taking into account the sequence similarity of *GLUD1P3* and GDH genes, such correlations may indicate an existing mechanism for the coordinated control of GDH mRNA and *GLUD1P3* RNA levels, which is further discussed. Importantly, correlations point only to the *GLUD1P3* gene, which is potentially involved in the regulation of GDH expression.

We also analyzed if the GDH pseudogenes are present in the ancient genomes. The sequences of *GLUD1P2–4* and *GLUD1P6–9* are available in Neanderthal and Denisovan genomes and the corresponding GRCh37.p13 (hg19) assembly of the human genome. This indicates no recent pseudogene appearance in the modern human genome. Of note, sequences of the spliced *GLUD1P3* RNA appeared to be 100% identical to modern human *GLUD1P3*.

## 3. Discussion

### 3.1. Impact of DNA Degradation on the Results

Here we analyzed evolution in *GLUD2* of ancient humans, localized the substitutions in protein structure and assessed their potential influence on clinical significance. However, substitutions in ancient DNA should be treated with caution since DNA is subjected to degradation and chemical modifications that can lead to postmortem mutations [39].

There are four different transitions often found in ancient DNA, which can be differentiated into two complementary groups according to their cause. Type 1 (TS1: A → G/T → C) is caused by deamination of adenine to hypoxanthine, and type 2 (TS2: C → T/G → A) is caused by deamination of cytosine or 5-methyl cytosine to uracil or thymine, respectively [22].

All studied substitutions have been evaluated for the potential effect of DNA degradation on the sequencing results. *GLUD2 Denisova 3* demonstrated a high number (10) of unique substitutions compared to other archaic human genomes. Eight of ten substitutions were more likely to occur due to DNA degradation and were ruled out of the analysis. Our estimation of the potential number of such transitions based on the *GLUD1* gene provides additional evidence that these transitions should be excluded. However, our exclusion criterion might be too strict, and some of these substitutions could be, in fact, caused by mutations of the Denisovan *GLUD2* gene.

No substitutions specific to Altai Neanderthal were found. On the other hand, both c.227G>A and c.1055A>G found independently in Altai Neanderthal and Denisovan *GLUD2,* belong to potential TS2 and TS1, respectively. They correspond to R76H and K352R amino acid substitutions discussed above; thus, there is a chance that these substitutions could also be caused by DNA degradation. However, independent identification in both genomes makes it an unlikely scenario. The c.1055A>G substitution is further supported by the genomic data from chimpanzees.

Thus, our analysis has revealed four new potential missense mutations in ancient human genomes, of which one (R76H) was found in Altai Neanderthal and Denisovan genomes and three more (T154P, I358L and S498A) were found in the Denisovan genome only. Importantly, all the latter three mutations are known as *GLUD2* variants present in modern human populations with no or little negative consequences, at least at a younger age, likely suggesting the Denisovan *GLUD2* variant to be also viable (Table 2).

### 3.2. Potential Role of Gene Flow

The phylogenetic tree of *GLUD2* coding sequences showed relatively low support and in general did not correspond to the evolution of archaic humans, which could be due to the low number of fixed mutations from the most recent common ancestor of humans and chimpanzees. There were four common mutations in modern and archaic humans, an additional seven unique mutations in *Denisova 3*, and one mutation in other *GLUD2* sequences. However, the tree enabled us to show that *GLUD2* sequences of younger Neanderthals *Chagyrskaya 8* and *Vindija 33.19* are identical to *GLUD2* of modern humans, although *H.sapiens* split from Neanderthals and Denisovans first [18]. Modern human, *Chagyrskaya 8*, and *Vindija 33.19* share one missense mutation c.1055G>A (R352K), in addition to four common mutations in all humans. Although possible, it is unlikely that this mutation could occur independently in humans and the common ancestor of *Chagyrskaya 8* and *Vindija 33.19.* Another option would be the gene flow between populations of humans and Neanderthals. To date, there is only one known SNP in the human population in this position c.1055A>C (dbSNP: rs2521651904), which occurs in the European population with low frequency (C = 0.000002) and has not been seen in other populations. The variant corresponds to the K352T substitution, which is different from a potential ancient-like K352R and c.1055A>G mutation not found in modern humans. Thus, all modern humans, including those populations that have never been in contact with Neanderthals, possess the mutated c.1055G>A variant. In contrast, only younger Neanderthals have this variant, meaning that the common ancestor of *Chagyrskaya 8* and *Vindija 33.19* could acquire this mutation through gene flow.

The *GLUD2* coding sequence of *Denisova 5* (Altai Neanderthal) was grouped with *Denisova 3* on the phylogenetic tree since they share one missense mutation c.227G>A (R76H). Denisovans split from Neanderthals approximately 400–440 kya, and *Denisova 5* split from younger Neanderthals approximately 130–145 kya. *Denisova 5* lived 40–60 kya earlier than *Denisova 3*. Thus, the occurrence of c.227G>A mutation either in ancestors of *Denisova 5* after the split from younger Neanderthals or in ancestors of *Denisova 3* after the split from Neanderthals with further gene flow is possible.

Importantly, analysis of the archaic genome data in addition to the genomic data of all modern apes [4] clarified the order of the last two missense mutations in human GDH2 evolution. The Leu468 was found in humans, Neanderthals and Denisovan included; thus, the M468L mutation occurred in the common ancestor of all humans. It preceded R352K substitution, which occurred further in ancestors of modern humans who may have shared it with younger Neanderthals through gene flow.

### 3.3. Structural Data on GDH2 Mutations Compared to Other Species

The *GLUD2* gene evolution has been a target of positive selection since its emergence [2]. Although the most striking mutations occurred soon after the gene duplication, many other ones were accumulated later in all *GLUD2*-possessing primate species, most likely as a response to destabilizing mutations such as ancestral R496S substitution [12,40] and also in accordance with the novel tissue-specific and cellular localization-specific functions [4]. The effect of such amino acid substitutions on intramolecular interactions has been recently evaluated using in silico predictions [40]. Most of these mutations can be classified into several subtypes according to their structural localization. The GDH2 missense mutations are often localized (1) in the proximity to the NAD(P)(H) site, (2) near the C-terminal site or Leu site, (3) within or close to the antenna, (4) within the hydrophobic core of the NAD domain, or (5) at the surface of the NAD domain [4]. Such localization likely corresponds to alterations in regulation by small ligands, protein-protein interactions, altered interactions between subunits and domains, and altered regulation by post-translational modifications. Novel mutations add to the same subtypes, localizing in the same sites as in other primates or close to them.

Particularly, the human genus-specific mutation M468L is localized to the antenna (Figure 3A), and is structurally close to the substitutions E492D in the *Gorilla*, or Q494R in the *Pongo*, or H481R in the *Nomascus*, or substitutions at positions 495–496 in *Hylobates moloch*. Their effect is not obvious and may be adaptive in its nature to previous substitutions, such as R496S and G509A occurring in common ancestors of all Hominoidea [4].

The GDH2 variants with arginine in position 352 are characteristic of all living Hominidae, such as orangutans, gorillas, and chimpanzees, with the exception of modern humans, who have lysine in this position [4]. Such a feature can also be found only in gibbons. Analysis of ancient DNA has revealed that Denisovan and Altai Neanderthal humans had Arg352, similarly to most Hominidae, but the Neanderthals *Chagyrskaya 8* and *Vindija 33.19* already had Lys352, similarly to modern humans. The difference between Arg352 and Lys352 is mainly in the ability for regulation by acylation, which is acquired by R352K substitution, probably independently in ancestors of modern humans and younger Neanderthals or due to the gene flow. The regulation is relevant for GDH2 NAD(H) or NADP(H) substrate specificity, as deacylated Lys352 forms an ionic bond with NADP(H), while the acylated form does not (Figure 3B).

The second substitution found both in Denisovan and Altai Neanderthal, namely R76H, is located at the protein surface and can be involved in GDH2 allosteric regulation, as shown above (Figure 3C,D). Importantly, the R76H substitution takes place next to the Gorilla-specific substitutions K415R and L418Q at the surface of the NAD domain [4]. Together with Arg76 and a few more residues, they form a small cavity that is well accessible to the solvent and could act as a binding center for ions like sodium (Na605, Na606 from GDH2, PDB ID: 6G2U, [27]). Substitutions within this area, such as R76H, reducing the large arginine residue for a smaller and less positive histidine, would increase the size of the cavity, providing more access for the cations or ligands comparable in size to amino acids or slightly larger. For example, this site could be a potential binding site for thiamine, as indicated by the binding pattern of thiamine or its derivatives found in the GDH sequence, corresponding to residues 412–414 of human GDH2 [41].

All the three Denisovan-specific substitutions are localized in the sites also known for mutations in other species. Position of the substitution T154P is the same as in the Hylobatidae genus, where alanine takes the place of threonine [4]. The mutations either in Denisovan or in gibbons eliminate phosphorylatable Thr154 residue close to the active site (Figure 3B). The other Denisovan-specific substitution, I358L, is present in chimpanzee genus [4]. Independent mutagenesis resulting in I358L substitution in GDH2 in Denisovan and chimpanzee can be proposed. Third Denisovan-specific substitution S498A is most known for its connection to Parkinson’s disease and potential protective role against Alzheimer’s disease in humans [4,29,42]. It is a gain-of-function mutation, resulting in enhanced GDH2 basal activity, lower GTP inhibition, and sensitization to estrogen inhibition [29]. No missense mutations are known in this position of GDH2 in other primate species, but a frequency of approximately 3% in the modern human population (Table 3) makes it one of the most frequent GDH2 variants despite its pathogenicity for older people.

Thus, structural localization of the GDH2 mutations found in Neanderthal and Denisovan genomes within the same or close to the mutation sites of GDH2 in other primate species strongly supports the viability of these new GDH2 variants.

### 3.4. GDH Pseudogenes and Their Potential Regulatory Role

The revealed correlations between expressions of mRNA of GDH1, GDH2, and *GLUD1P3* RNA in tissues (Figure 4) are most likely a consequence of a complex regulation of GDH expression. Such involvement of pseudogenes-encoded RNA in the expression of parental gene is known and can occur in multiple ways involving either sense or antisense sequences [43]. Other miRNAs are often involved in such regulation as well.

**Figure 5:**
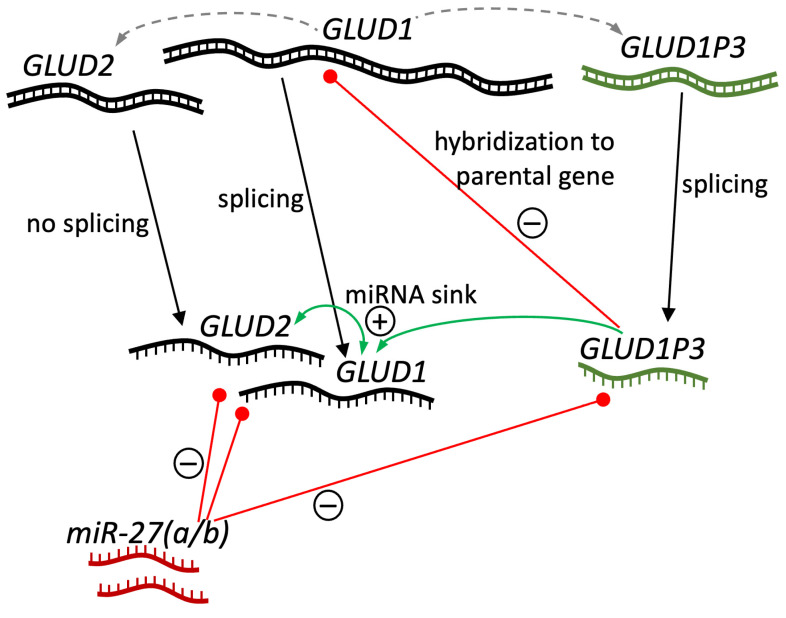
Expression regulation of GDH and its pseudogene(s). Expression of *GLUD1*, *GLUD2* and *GLUD1P3* pseudogene, their potential interaction between each other and with miR-27 are depicted. Other GDH pseudogenes are not included, as no correlations of their expression and expression of protein-coding GDH genes have been revealed. DNA and RNA are depicted as double-stranded and single-stranded lines, respectively. *GLUD1P3* DNA/RNA are of dark green color, miR-27a and miR27b RNA are colored in dark red. *GLUD2* and *GLUD1P3* originating from *GLUD1* are shown as grey dashed arrows. Difference in the presence of splicing is specified on the figure. Negative regulation of expression either by miRNA or by hybridization to parental DNA is shown as red lines with “minus” signs. “Plus” sign and green arrows indicate positive regulation between RNAs likely acting as miRNA sinks to each other.

Previously, repression of GDH2 by miR-27a has been proposed [44,45]. Moreover, repression of intronless genes is common for most tissues [46], and such regulation may substantially influence the distribution of GDH2 in the human body [4]. Since GDH1 and GDH2 mRNA are very similar to each other due to GDH2 originating from GDH1, both of them are most likely involved in potential miRNA repression. In other words, they may act as miRNA sinks for each other (Figure 5).

Based on the available data indicating miR-27a’s role in *GLUD2* repression [44,45], we compared the sequences of miR-27a as well as miR-27b and RNAs of *GLUD1*, *GLUD2,* and *GLUD1P3*. The sequence of miR-27b revealed high sequence identity to miR-27a (68% identity, 54 of 79 nucleotides in Smith–Waterman local alignment), both having a 32 nt-long mostly identical (26 of 32 nt) subsequence near the 5′-end, and a completely identical 20 nt-long subsequence close to the 3′-end. Such sequence similarity may be of regulatory significance for potential RNA interference with miRNA targets, as 20 nt may be enough for silencing of the same targets.

A 54% identity of miR-27b to the reverse complement sequence of GDH1 mRNA was revealed (54 of 100 nt in Smith–Waterman local alignment). The full sequence of miR-27b was aligned to the GDH1 reverse complement sequence with no big gaps. The matching part corresponds to the 1st and 2nd exons of GDH1. The sequence matching miR-27b is also completely identical between GDH1 and GDH2 mRNA. Such an imperfect but long match may indicate potential GDH1 and GDH2 silencing by miR-27b in cells or tissues with substantial miR-27b expression.

Although miR-27a has revealed less identity to the reverse complement sequence of GDH1 mRNA, including both the full-length reverse complement sequence (42% identity, 53 of 125 nt) and the area matching miR-27b (40% identity, 61 of 154 nt), still a 33 nt-long area with 24 identical nt could be detected. Thus, miR-27 (a and b) can be the RNA involved in silencing GDH1 and GDH2, which in turn can act as a sink for miR-27, supporting each other’s expression (Figure 5). The latter may lead to the revealed positive correlation of GDH1 and GDH2 mRNA in human tissues (Figure 4A).

Analysis of miR-27 alignments with *GLUD1P3* spliced RNA reverse complement sequence has revealed comparable percent identity for miR-27a and miR-27b—44% (42 of 95 nt) and 45% (68 of 150 nt), respectively. Comparison to *FOXO1,* which is regulated by these miRNAs [47], was performed as a positive control. The identity of miR-27a and miR-27b to the *FOXO1* reverse complement sequence was 48% (59 of 124 nt) and 44% (66 of 149 nt), respectively. With that in mind, we can propose that both miR-27a and miR-27b can pair with *GLUD1P3* RNA. Thus, according to sequence analysis, *GLUD1P3* RNA could act as a sink for the miR-27 miRNAs silencing GDH1 and/or GDH2 (Figure 5). Of note, *GLUD1P3* expression at approximately a 1% ratio compared to *GLUD1* levels is enough for such regulation, and a significant dose-dependent effect on parental gene transcript concentration of such a miRNA sink can be achieved [43,48].

Yet, another mechanism is most likely responsible for the negative correlation of *GLUD1P3* and GDH1 expression (Figure 4B). Although correlation does not imply causation, a possible mechanism explaining such a link is known. That is, an RNA transcribed from a pseudogene can epigenetically target the parental gene DNA and suppress its transcription (Figure 5) [43]. For example, a *PTEN* pseudogene antisense RNA suppressed *PTEN* expression by recruiting the chromatin-repressor proteins EZH2 and DNMT3A to the *PTEN* promoter [49]. Independent appearance of *GLUD2* and *GLUD1P3* from *GLUD1*, and significance of correlation between *GLUD1P3* and *GLUD1* with only a trend (*p* = 0.078) for *GLUD1P3* and *GLUD2,* suggests *GLUD1* may be a preferred target of *GLUD1P3* (Figure 5).

## 4. Materials and Methods

### 4.1. Sequences of GDH Genes and Pseudogenes

mRNA sequences of *GLUD1* and *GLUD2* genes from primates were downloaded from RefSeq database [50]. For ancient humans, genomes with high coverage (~30×) were chosen for the analysis (Table S1). The vcf files with genetic variants in ancient human genomes were downloaded from http://cdna.eva.mpg.de/denisova, http://cdna.eva.mpg.de/neandertal (accessed on 12 April 2025). The consensus sequences of *GLUD2*, *GLUD1* and GDH pseudogenes in ancient human genomes were obtained using samtools and bcftools version 1.21 [51]. The introns and non-translated regions in *GLUD1* and *GLUD1P3* sequences were cut according to transcript variant 1 of human *GLUD1* mRNA (NM_005271).

Sequences were aligned using MAFFT software version 7 [52]. The alignments were visualized in AliView software version 1.30 [26].

### 4.2. Phylogenetics Analysis

The phylogenetic tree for *GLUD2* mRNA of primates and ancient and modern humans, was inferred using the IQ-TREE v2.0.6 program [53] incorporating the best-fit nucleotide substitution model (K2P + R2) [54]. The number of ultrafast bootstrap replicates was 10,000 [55].

The ancestral sequence was inferred using the maximum likelihood method [56] under the Kimura 2-parameter model [24] implemented in MEGA11 [25]. The analysis involved coding sequences of *GLUD1* and *GLUD2* from apes, including archaic and modern humans. The phylogenetic tree was visualized in FigTree (https://tree.bio.ed.ac.uk/software/figtree/) (accessed on 12 April 2025).

### 4.3. Structural Analysis of GDH Mutations

PyMOL v2.5 (PyMOL Molecular Graphics System, Schrödinger, LLC, New York, NY, USA) was used for visualization of GDH structure. Protein subunits were shown as cartoon models. The models of structures are obtained from the Protein Data Bank (PDB). Structural alignment (“align”) of the proteins by PyMOL v2.5 is used for comparison of the two GDH structures with different ligands. Residue numbers are written according to the full-length sequence.

### 4.4. Analysis of Available Neanderthal-like GDH2 Variants and Their Clinical Significance

Missense variants of GDH (*GLUD1* or *GLUD2* genes) are observed in patients. Data from the ClinVar [33], COSMIC [34], NCBI dbSNP [35], Ensembl [36], gnomAD [37] and Uniprot [38] databases were used. The medical condition was written as submitted to the corresponding database.

### 4.5. Correlation Analysis of (Pseudo)Gene Expression

Matrices of read counts for bulk RNA sequencing experiments in human tissues were downloaded from GTEx V8 Portal on 27 October 2025. The data from cultured cells (“Cells—Cultured fibroblasts” and “Cells—Leukemia cell line (CML)”) were omitted. Spearman correlations between TPM expressions of *GLUD1*, its pseudogenes, and *GLUD2* were calculated and visualized using scipy [57] and matplotlib [58] Python 3.10 libraries. Correlation coefficients and *p*-values are mentioned as “r” and “*p*”, respectively.

### 4.6. Sequence Alignments of MiR-27, GLUD1, GLUD2, and GLUD1P3

Evaluation of potential binding sites were performed by local pairwise sequence alignments using Smith–Waterman algorithm [59].

## Figures and Tables

**Table 1:** *GLUD1* substitutions in the Denisovan sequence (*Denisova 3*), according to the multiple sequence alignment (Figure S1). The transition type is specified according to its potential deamination cause as follows. TS1: A → G/T → C and TS2: C → T/G → A. Nine out of ten found substitutions that belong either to TS1 or TS2, pointing to their cause by DNA degradation, especially taking into account high conservation of the *GLUD1* gene due to the purifying selection [2].

Substitution	Transition Type	Potential Cause
c.342T>C	TS1	DNA degradation
c.376G>A	TS2	DNA degradation
c.462T>C	TS1	DNA degradation
c.527T>C	TS1	DNA degradation
c.771T>C	TS1	DNA degradation
c.909A>C	none	synonymous mutation
c.942A>G	TS1	DNA degradation
c.1175A>G	TS1	DNA degradation
c.1255G>A	TS2	DNA degradation
c.1479G>A	TS2	DNA degradation

**Table 2:** *GLUD2* substitutions in the Denisovan sequence (*Denisova 3*) that do not correspond to the ancestral genome according to the multiple sequence alignment (Figure S2). The transition type is specified according to its potential deamination cause as follows. TS1: A → G/T → C and TS2: C → T/G → A. Eight out of thirteen found substitutions belong either to TS1 or TS2, which suggests them to be caused by DNA degradation.

Substitution	Transition Type	Potential Cause	Synonymous or Not
c.94C>A	none	mutation	synonymous
c.103G>A	TS2	DNA degradation	G35R
c.123G>T	none	mutation	synonymous
c.232G>A	TS2	DNA degradation	A78T
c.460A>C	none	mutation	T154P
c.582C>T	TS2	DNA degradation	synonymous
c.986C>T	TS2	DNA degradation	A329V
c.1072A>C	none	mutation	I358L
c.1184G>A	TS2	DNA degradation	R395K
c.1241A>G	TS1	DNA degradation	D414G
c.1371A>G	TS1	DNA degradation	synonymous
c.1395C>T	TS2	DNA degradation	synonymous
c.1492T>G	none	mutation	S498A

## Data Availability

The presented data are available in this article and Supplementary Materials.

## Supplementary Materials

The supporting information can be downloaded at: https://www.mdpi.com/article/10.3390/ijms26094322/s1.

## Abbreviations

GDH	glutamate dehydrogenase
ky	1000 years
kya	1000 years ago
TS1	transition type 2
TS2	transition type 2
GTEx	Genotype-Tissue Expression
