# Supplementary material for: Neanderthal and Denisovan Glutamate Dehydrogenase 2 Evolution and Clinical Significance
Source: Int J Mol Sci. 2025 May 1;26(9):4322. doi: 10.3390/ijms26094322 (PMC12072557; doi:10.3390/ijms26094322)
Supplement: Supplementary file 1 [file ijms-26-04322-s001.zip › SupplementaryMaterials2.pdf]

Supplementary Materials

Neandertal and Denisovan Glutamate Dehydrogenase 2 Evolution and Clinical Significance

Yulia A. Aleshina, Lev G. Zavileyskiy and Vasily A. Aleshin

Table S1. The genomes of ancient humans used for the analysis of GDH genes and pseudogenes.

Genome of ancient human	Species	Location	Reference	URL
Denisova 3	*Homo sapiens subsp. denisova*	Denisova Cave, Russia	(Meyer, Kircher et al. 2012)	http://cdna.eva.mpg.de/denisova/VCF/hg19_1000g/
Vindija 33.19	*Homo neanderthalensis*	Vindija Cave, Croatia	(Prufer, de Filippo et al. 2017)	http://cdna.eva.mpg.de/neandertal/Vindija/VCF/indels/Vindija33.19_chrALL_indels
Chagyrskaya 8	*Homo neanderthalensis*	Chagyrskaya Cave, Russia	(Mafessoni, Grote et al. 2020)	http://cdna.eva.mpg.de/neandertal/Chagyrskaya/VCF/
Denisova 5/Altai Neandertal	*Homo neanderthalensis*	Denisova Cave, Russia	(Prüfer, Racimo et al. 2013)	neandertal/altai/Al http://cdna.eva.mpg.de/taiNeandertal/VCF/

Table S2. Availability of the missense GDH2 variants similar to Neandertal or Denisovan GDH2 in modern humans. Data are provided according to gnomAD (Chen, Francioli et al. 2023). N.D. – no data.

	**Allele Frequencies**		
**Genetic Ancestry Group**	**T154P**	**I358L**	**S498A**
African/African American	0.004413	0	0.05254
Middle Eastern	0	0	0.04786
South Asian	0.00001761	0	0.04742
European (Finnish)	0	0	0.03642
Remaining	0.0001681	0	0.02870
European (non-Finnish)	0	0	0.02420
Ashkenazi Jewish	0	0	0.01934
Admixed American	0.0001741	0	0.01314
Amish	0	0	0.01016
East Asian	0	0.00002962	0.00005922
XX	0.0002474	0.000001231	0.02570
XY	0.0001734	0	0.02714
**Total**	**0.0002231**	**8.268e-7**	**0.02618**
Daghestan*	N.D.	N.D.	0.095

* According to "**Extensive genome-wide autozygosity in the population isolates of Daghestan**" dbSNP dataset (Karafet, Bulayeva et al. 2015).

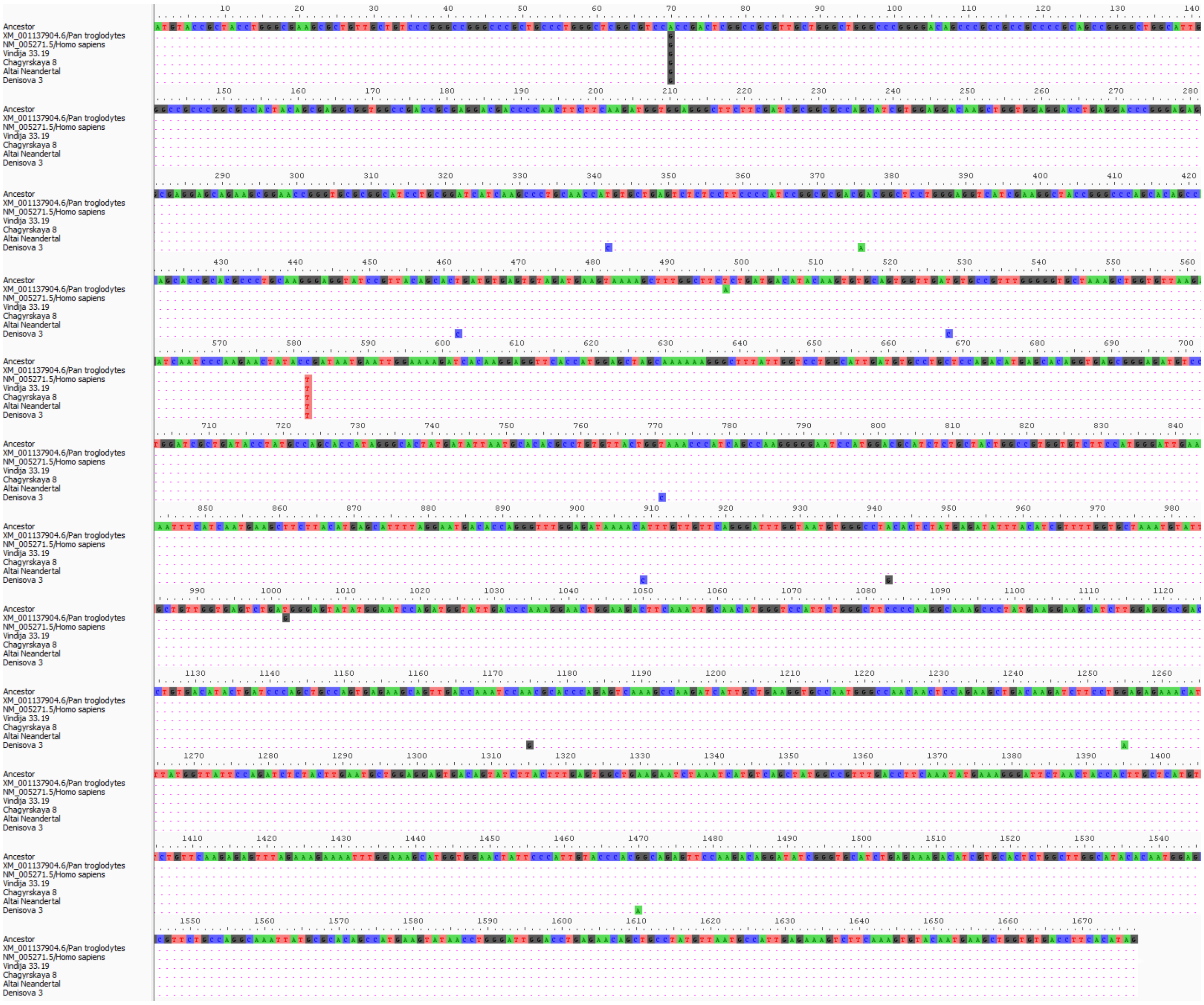

Figure S1. Multiple sequence alignment for *GLUD1* mRNA sequences from chimpanzee, modern and archaic humans (Vindija 33.19, Chagyrskaya 8, Altai Neandertal, Denisova 3). The ancestral sequence was inferred using the maximum likelihood method (Nei and Kumar 2000) under the Kimura 2-parameter model (Kimura 1980) implemented in MEGA11 (Tamura, Stecher and Kumar 2021). Dots indicate nucleotides that are identical to the ancestral sequence. The alignment was visualized in AliView v1.28 software (Larsson 2014).

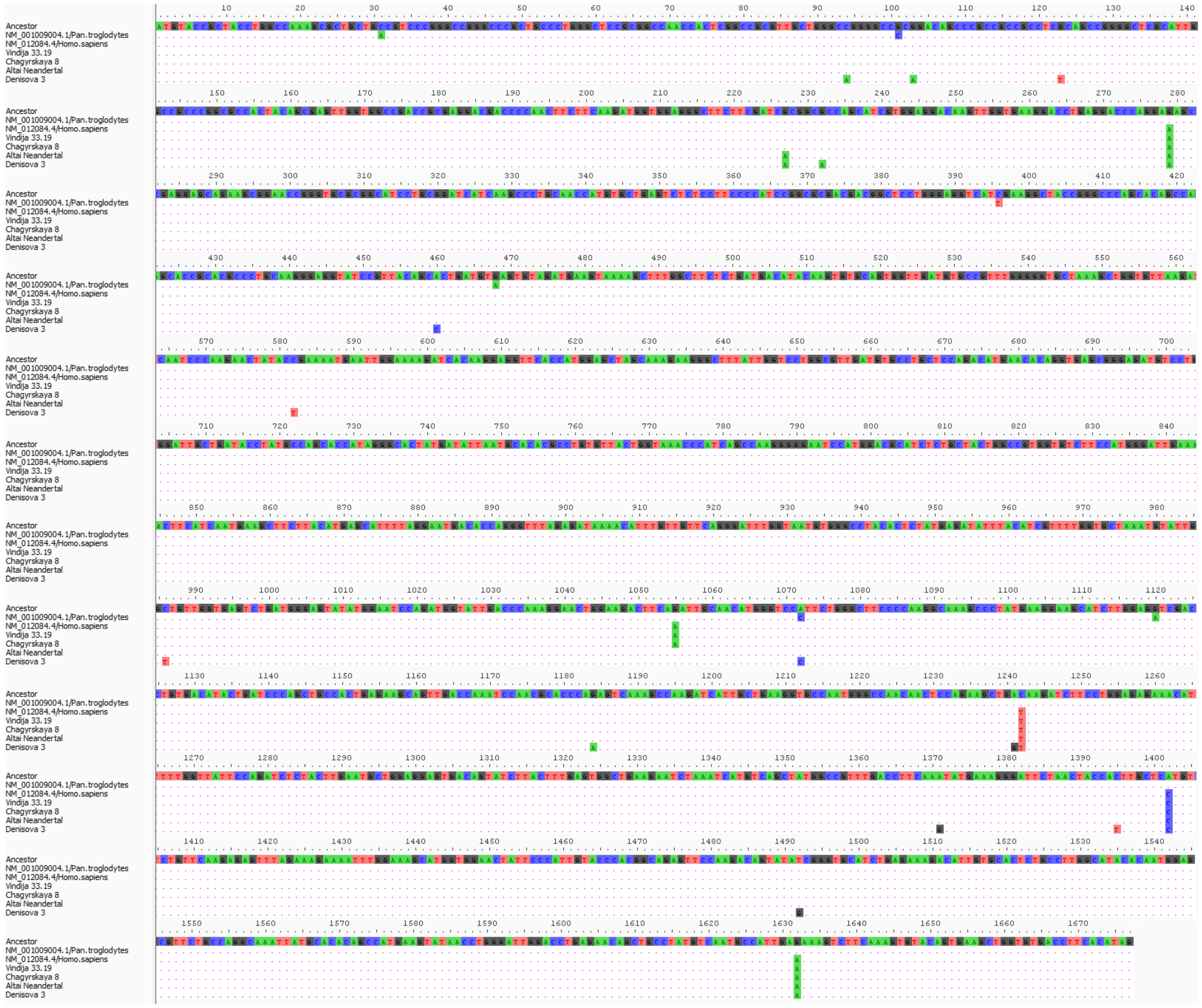

Figure S2. Multiple sequence alignment for *GLUD2* mRNA sequences from chimpanzee, modern and archaic humans (Vindija 33.19, Chagyrskaya 8, Altai Neandertal, Denisova 3). The ancestral sequence was inferred using the maximum likelihood method (Nei and Kumar 2000) under the Kimura 2-parameter model (Kimura 1980) implemented in MEGA11 (Tamura, Stecher and Kumar 2021). Dots indicate nucleotides that are identical to the ancestral sequence. The alignment was visualized in AliView v1.28 software (Larsson 2014).

Chen, S., L. C. Francioli, J. K. Goodrich, R. L. Collins, M. Kanai, Q. Wang, J. Alföldi, N. A. Watts, C. Vittal, L. D. Gauthier, T. Poterba, M. W. Wilson, Y. Tarasova, W. Phu, R. Grant, M. T. Yohannes, Z. Koenig, Y. Farjoun, E. Banks, S. Donnelly, S. Gabriel, N. Gupta, S. Ferriera, C. Tolonen, S. Novod, L. Bergelson, D. Roazen, V. Ruano-Rubio, M. Covarrubias, C. Llanwarne, N. Petrillo, G. Wade, T. Jeandet, R. Munshi, K. Tibbetts, M. Abreu, C. A. Aguilar Salinas, T. Ahmad, C. M. Albert, D. Ardissino, I. M. Armean, E. G. Atkinson, G. Atzmon, J. Barnard, S. M. Baxter, L. Beaugerie, E. J. Benjamin, D. Benjamin, M. Boehnke, L. L. Bonnycastle, E. P. Bottinger, D. W. Bowden, M. J. Bown, H. Brand, S. Brant, T. Brookings, S. Bryant, S. E. Calvo, H. Campos, J. C. Chambers, J. C. Chan, K. R. Chao, S. Chapman, D. I. Chasman, R. Chisholm, J. Cho, R. Chowdhury, M. K. Chung, W. K. Chung, K. Cibulskis, B. Cohen, K. M. Connolly, A. Correa, B. B. Cummings, D. Dabelea, J. Danesh, D. Darbar, P. Darnowsky, J. Denny, R. Duggirala, J. Dupuis, P. T. Ellinor, R. Elosua, J. Emery, E. England, J. Erdmann, T. Esko, E. Evangelista, D. Fatkin, J. Florez, A. Franke, J. Fu, M. Färkkilä, K. Garimella, J. Gentry, G. Getz, D. C. Glahn, B. Glaser, S. J. Glatt, D. Goldstein, C. Gonzalez, L. Groop, S. Gudmundsson, A. Haessly, C. Haiman, I. Hall, C. L. Hanis, M. Harms, M. Hiltunen, M. M.

Holi, C. M. Hultman, C. Jalas, M. Kallela, D. Kaplan, J. Kaprio, S. Kathiresan, E. E. Kenny, B.-J. Kim, Y. J. Kim, D. King, G. Kirov, J. Kooner, S. Koskinen, H. M. Krumholz, S. Kugathasan, S. H. Kwak, M. Laakso, N. Lake, T. Langsford, K. M. Laricchia, T. Lehtimäki, M. Lek, E. Lipscomb, R. J. F. Loos, W. Lu, S. A. Lubitz, T. T. Luna, R. C. W. Ma, G. M. Marcus, J. Marrugat, K. M. Mattila, S. McCarroll, M. I. McCarthy, J. L. McCauley, D. McGovern, R. McPherson, J. B. Meigs, O. Melander, A. Metspalu, D. Meyers, E. V. Minikel, B. D. Mitchell, V. K. Mootha, A. Naheed, S. Nazarian, P. M. Nilsson, M. C. O'Donovan, Y. Okada, D. Ongur, L. Orozco, M. J. Owen, C. Palmer, N. D. Palmer, A. Palotie, K. S. Park, C. Pato, A. E. Pulver, D. Rader, N. Rahman, A. Reiner, A. M. Remes, D. Rhodes, S. Rich, J. D. Rioux, S. Ripatti, D. M. Roden, J. I. Rotter, N. Sahakian, D. Saleheen, V. Salomaa, A. Saltzman, N. J. Samani, K. E. Samocha, A. Sanchis-Juan, J. Scharf, M. Schleicher, H. Schunkert, S. Schönherr, E. G. Seaby, S. H. Shah, M. Shand, T. Sharpe, M. B. Shoemaker, T. Shyong, E. K. Silverman, M. Singer-Berk, P. Sklar, J. T. Smith, J. G. Smith, H. Soininen, H. Sokol, R. G. Son, J. Soto, T. Spector, C. Stevens, N. O. Stitziel, P. F. Sullivan, J. Suvisaari, E. S. Tai, K. D. Taylor, Y. Y. Teo, M. Tsuang, T. Tuomi, D. Turner, T. Tusie-Luna, E. Vartiainen, M. Vawter, L. Wang, A. Wang, J. S. Ware, H. Watkins, R. K. Weersma, B. Weisburd, M. Wessman, N. Whiffin, J. G. Wilson, R. J. Xavier, A. O'Donnell-Luria, M. Solomonson, C. Seed, A. R. Martin, M. E. Talkowski, H. L. Rehm, M. J. Daly, G. Tiao, B. M. Neale, D. G. MacArthur and K. J. Karczewski (2023). "A genomic mutational constraint map using variation in 76,156 human genomes." Nature **625**(7993): 92-100.

Karafet, T. M., K. B. Bulayeva, O. A. Bulayev, F. Gurgenova, J. Omarova, L. Yepiskoposyan, O. V. Savina, K. R. Veeramah and M. F. Hammer (2015). "Extensive genome-wide autozygosity in the population isolates of Daghestan." European Journal of Human Genetics **23**(10): 1405-1412.

Kimura, M. (1980). "A simple method for estimating evolutionary rates of base substitutions through comparative studies of nucleotide sequences." J Mol Evol **16**(2): 111-120.

Larsson, A. (2014). "AliView: a fast and lightweight alignment viewer and editor for large datasets." Bioinformatics **30**(22): 3276-3278.

Mafessoni, F., S. Grote, C. de Filippo, V. Slon, K. A. Kolobova, B. Viola, S. V. Markin, M. Chintalapati, S. Peyrégne, L. Skov, P. Skoglund, A. I. Krivoshapkin, A. P. Derevianko, M. Meyer, J. Kelso, B. Peter, K. Prüfer and S. Pääbo (2020). "A high-coverage Neandertal genome from Chagyrskaya Cave." Proceedings of the National Academy of Sciences **117**(26): 15132-15136.

Meyer, M., M. Kircher, M.-T. Gansauge, H. Li, F. Racimo, S. Mallick, J. G. Schraiber, F. Jay, K. Prüfer, C. de Filippo, P. H. Sudmant, C. Alkan, Q. Fu, R. Do, N. Rohland, A. Tandon, M. Siebauer, R. E. Green, K. Bryc, A. W. Briggs, U. Stenzel, J. Dabney, J. Shendure, J. Kitzman, M. F. Hammer, M. V. Shunkov, A. P. Derevianko, N. Patterson, A. M. Andrés, E. E. Eichler, M. Slatkin, D. Reich, J. Kelso and S. Pääbo (2012). "A High-Coverage Genome Sequence from an Archaic Denisovan Individual." Science **338**(6104): 222-226.

Nei, M. and S. Kumar (2000). Molecular Evolution and Phylogenetics.

Prufer, K., C. de Filippo, S. Grote, F. Mafessoni, P. Korlevic, M. Hajdinjak, B. Vernot, L. Skov, P. Hsieh, S. Peyregne, D. Reher, C. Hopfe, S. Nagel, T. Maricic, Q. Fu, C. Theunert, R. Rogers, P. Skoglund, M. Chintalapati, M. Dannemann, B. J. Nelson, F. M. Key, P. Rudan, Z. Kucan, I. Gusic, L. V. Golovanova, V. B. Doronichev, N. Patterson, D. Reich, E. E. Eichler, M. Slatkin, M. H. Schierup, A. M. Andres, J. Kelso, M. Meyer and S. Paabo (2017). "A high-coverage Neandertal genome from Vindija Cave in Croatia." Science **358**(6363): 655-658.

Prüfer, K., F. Racimo, N. Patterson, F. Jay, S. Sankararaman, S. Sawyer, A. Heinze, G. Renaud, P. H. Sudmant, C. de Filippo, H. Li, S. Mallick, M. Dannemann, Q. Fu, M. Kircher, M. Kuhlwilm, M. Lachmann, M. Meyer, M. Ongyerth, M. Siebauer, C. Theunert, A. Tandon, P. Moorjani, J. Pickrell, J. C. Mullikin, S. H. Vohr, R. E. Green, I. Hellmann, P. L. F. Johnson, H. Blanche, H. Cann, J. O. Kitzman, J. Shendure, E. E. Eichler, E. S. Lein, T. E. Bakken, L. V. Golovanova, V. B. Doronichev, M. V. Shunkov, A. P. Derevianko, B. Viola, M. Slatkin, D. Reich, J. Kelso and S. Pääbo (2013). "The complete genome sequence of a Neanderthal from the Altai Mountains." Nature **505**(7481): 43-49.

Tamura, K., G. Stecher and S. Kumar (2021). "MEGA11: Molecular Evolutionary Genetics Analysis Version 11." Mol Biol Evol **38**(7): 3022-3027.
